# Quantifying DNA damage induced by ionizing radiation and hyperthermia using single DNA molecule imaging

**DOI:** 10.1016/j.tranon.2020.100822

**Published:** 2020-07-08

**Authors:** Vandana Singh, Pegah Johansson, Dmitry Torchinsky, Yii-Lih Lin, Robin Öz, Yuval Ebenstein, Ola Hammarsten, Fredrik Westerlund

**Affiliations:** a Biology and Biological Engineering, Chalmers University of Technology, Gothenburg, Sweden; b Laboratory of Clinical Chemistry, Sahlgrenska University Hospital, Gothenburg, Sweden; c Department of Laboratory Medicine, Institute of Biomedicine, Sahlgrenska Academy at University of Gothenburg, Gothenburg, Sweden; d Raymond and Beverly Sackler Faculty of Exact Sciences, School of Chemistry, Tel Aviv University, Israel

**Keywords:** SSB, single-strand break, DSB, double-strand break, IR, ionizing radiation, APE1, apurinic/apyrimidinic endonuclease 1, BER, base excision repair, PBMCs, peripheral blood mononuclear cells

## Abstract

Ionizing radiation (IR) is a common mode of cancer therapy, where DNA damage is the major reason of cell death. Here, we use an assay based on fluorescence imaging of single damaged DNA molecules isolated from radiated lymphocytes, to quantify IR induced DNA damage. The assay uses a cocktail of DNA-repair enzymes that recognizes and excises DNA lesions and then a polymerase and a ligase incorporate fluorescent nucleotides at the damage sites, resulting in a fluorescent “spot” at each site. The individual fluorescent spots can then be counted along single stretched DNA molecules and the global level of DNA damage can be quantified. Our results demonstrate that inclusion of the human apurinic/apyrimidinic endonuclease 1 (APE1) in the enzyme cocktail increases the sensitivity of the assay for detection of IR induced damage significantly. This optimized assay also allowed detection of a cooperative increase in DNA damage when IR was combined with mild hyperthermia, which is sometimes used as an adjuvant in IR therapy. Finally, we discuss how the method may be used to identify patients that are sensitive to IR and other types of DNA damaging agents.

## Introduction

Most cancer patients are treated with radiotherapy at some stage [1,2]. Radiotherapy kills cancer cells by inducing DNA damage [3], but the normal tissue toxicity in response to radiotherapy is highly variable among patients [4]. The doses used today sometimes result in long-term side effects such as fibrosis and severe gastrointestinal problems, likely because the sensitive minority is overdosed [5]. The mechanisms behind the well-known variations in long-term side effects in patients that received the same dose is not well understood and assays to identify sensitive patients are highly sought for.

Radiotherapy uses ionizing radiation (IR) generated by different sources, such as X- rays, γ- radiation, electron, proton and neutron beams [6]. IR induces many types of DNA damage, including single strand breaks (SSBs), pyrimidine lesions and purine lesions [7,8]. The passage of IR through the cell nucleus often results in the formation of a variety of lesions within one or two helical turns of the DNA resulting in DSBs, SSBs with opposing base damage or other combinations [9]. These complex DNA lesions are referred to as clustered DNA damage [8,9]. Clustered damage sites are highly repair resistant because no undamaged strand is present to guide the DNA-repair machinery resulting in effective cell killing [10]. While the mechanism of IR induced damage in cell lines has been well studied [11], the ability to investigate the variation in DNA-repair in normal tissue from different individuals may shed light on the mechanism responsible for this variation. This may allow the development of strategies to individualize IR treatment dosing.

Hyperthermia is making its way into the clinic as an adjuvant to conventional cancer therapy [12] and is sometimes used as a sensitizer to improve the efficacy of IR [[13], [14], [15], [16]]. In these cases, the tissue is exposed to temperatures of up to 45 °C [17], which inhibits repair of IR induced DNA breaks [[18], [19], [20]]. Several mechanisms for hyperthermia induced cell death have been proposed including oxidative stress, induction of endonucleases, degradation of topoisomerase I and topoisomerase II [[21], [22], [23]], and inhibition of DNA glycosylases and excision repair pathway enzymes [22,24]. Furthermore, some studies have reported that hyperthermia causes induction of DNA breaks on its own, possibly due to increased levels of 8-oxo guanine, apurinic sites, and deaminated cytosines [25,26].

Methods used to asses DNA damage include comet assay [27], DNA breakage detection-FISH [28], radioimmunoassay [29], enzyme-linked immunosorbent assay [30], terminal deoxynucleotidyl transferase dUTP nick-end labeling assay [31], ligation mediated PCR [32], and electrochemical detection [30]. Although, most methods focus on DSB quantification [33], some techniques are available also for quantifying SSBs. However, these methods in general only allow characterization of one type of damage at a time, while in many cases it is more relevant to investigate the total amount of DNA damage.

Recently, an assay for quantifying single stranded DNA damage, based on single DNA molecule imaging, has been demonstrated [34,35]. Zirkin et al. used a commercial mix of DNA repair enzymes consisting of FpG, Endo IV, T4 PDG, *Bst* DNA Polymerase, Endo VIII, UDG, T4 PDG and *Taq* DNA Ligase, to incorporate fluorescent nucleotides, in an *in vitro* repair reaction, at the single-strand lesions [36]. The labeled DNA molecules were then stretched on activated coverslips and visualized using a fluorescence microscope so that the repaired sites appeared as fluorescent spots along the countour of the DNA molecules [34]. Using this assay, the repair dynamics in response to UV irradiation and reactive oxygen species was demonstrated and the effect of a defective DNA repair machinery on UV-induced lesions could be investigated [[34], [35], [36]].

One of the pathways to repair DNA damage induced by IR is base excision repair (BER) [37]. Repair of some IR induced DNA damage types is initiated by various glycosylases, followed by the action of apurinic/apyrimidinic (APE) endonucleases [38]. The human APE1 enzyme is homologous to exonuclease III (Exo III) in E.*coli* and is a key player of the BER pathway involved in the repair of reactive oxygen species induced DNA damage [39]. Exo III is needed for *in vitro* repair of 3′-phosphoglycolate lesions generated by γ-radiation [40]. The 3′-phosphoglycolatase activity of Exo III is four-fold higher than for endonuclease IV for the same AP endonuclease activity *in vitro* in the presence of Mg^2+^ [41]. *In vitro* rejoining of nicked mismatched DNA is dependent on APE1 [42]. APE1 antisense cells [43] and APE1 null blastocysts [44,45] have increased susceptibility towards IR, indicating the importance of APE1 in repair of IR induced DNA lesions.

Here, the method developed by Zirkin et al. was adapted for detecting single-strand lesions induced by IR and hyperthermia [36]. The study was performed on peripheral blood mononuclear cells (PBMCs) from healthy individuals. We used a cocktail of enzymes with different repair profiles as summarized in Table 1. APE1 was shown to significantly increase the amount of detected DNA damage induced by IR, in particular at higher IR doses. The assay also detected DNA damage in PBMCs treated with hyperthermia alone and showed hindered repair of IR induced DNA damage in PBMCs treated with hyperthermia.

**Table 1:** Details of DNA lesions detected by constituent of enzymatic cocktail used and the termini of the strand breaks formed after the action of the respective enzymes.

Repair enzyme	DNA substrates for enzyme action	Termini of the strand breaks induced by the enzyme action
FpG	8-oxo-7,8- dihydroguanine 2,6-diamino-4-hydroxy-5-formamidopyrimidine	5′-phosphate 3′-phosphate
Endo III	5-hydroxy-5-methylhydantoin thymine glycol 5-hydroxy-6-hydrothymine uracil glycol 5-hydroxy-6-hydrouracil	3′-α, β-unsaturated aldehyde 5′-phosphate
Endo IV	AP site 3′-phosphate 3′-phosphoglycolate	dR5P OH
Endo VIII	urea thymine glycol 5-hydroxy-5-methylhydantoin uracil glycol 6-hydroxy-5, 6-dihydrothymine	5′-phosphate 3′-phosphate
APE1	AP site 3′-phosphate 3′-phosphoglycolate	dR5P OH

## Materials and methods

### Collection of blood samples

Blood samples from apparently healthy individuals with normal differential blood count were collected from the Hematology unit at the Clinical Chemistry Department at Sahlgrenska University Hospital. PBMCs were isolated from blood samples through density centrifugation using Lymphoprep (Axis-Shield PoC AS, Oslo, Norway) according to the manufacturers' instructions. The study has been approved by the Regional Ethical Review Board (Dnr: 246-07 and Dnr: 308-08).

### Ionizing radiation treatment

PBMCs were resuspended in RPMI-1640 (Sigma-Aldrich) at 20 × 10^6^ cells/ml before treatment and 200 μl was used per ionizing radiation treatment. The cells were irradiated on ice in a 35 mm × 10 mm petridish without the lid, in an RS 2000 X-ray Irradiator (Rad Source Technologies) at the doses indicated in each figure. For repair kinetic studies, the samples were incubated in a water bath at 37 °C for the time intervals indicated in each figure after ionizing radiation treatment of 2 Gy.

Immediately after ionizing radiation treatment, 100 μl of ice-cold cell lysis buffer (0.5% Triton X-100, 0.004 M Tris-HCl, pH 7.4, 2 M NaCl) was added to stop any enzymatic processes. For hyperthermia treatments, the cells were incubated on the thermal block at temperatures ranging from 37 °C to 42 °C for the indicated periods of time. After hyperthermia treatment, the cells were either lysed or incubated at 37 °C for 30 min before lysis. For the combination of hyperthermia and IR, the samples were pre-treated at 42 °C for 30 min and then exposed to a 2 Gy dose of IR on ice. After hyperthermia-IR treatment the cells were either lysed or incubated at 37 °C for a further 5 or 30 min as indicated in the figure.

### Extraction of DNA

DNA was extracted using the GenElute-Mammalian Genomic DNA Miniprep Kit (Sigma-Aldrich), according to the company protocol with some modifications. The samples were not vortexed and wide bore tips were used to pipette the samples in order to maintain the integrity of genomic DNA. The proteinase K treatment was done at 55 °C for 20 min. 10 mM Tris-Cl, pH 8.5 was used for elution.

### Fluorescent labelling of DNA damage sites

Samples containing 500 ng of DNA were incubated with APE1 (5 U), FpG (1.6 U), Endo III (2 U), Endo IV (2 U) and Endo VIII (2 U) in the reaction buffer (0.05 M Tris HCl (pH 7.8), 0.1 mM dithiothreitol, 0.01 M MgSO_4_, 50 μg/ml bovine serum albumin) for 2 h at 37 °C in a final volume of 100 μl. The enzymes were inactivated at 65 °C for 5 min, followed by 4 h incubation at 16 °C with dNTPs (1 μM of dATP, dGTP, dCTP, 0.1 μM dTTP (Sigma-Aldrich) and 0.1 μM Aminoallyl-dUTP-ATTO-647 N (Jena Bioscience), in nick translation buffer (0.05 M Tris HCl (pH 7.5), 0.1 mM dithiothreitol, 0.01 M MgSO_4_, 50 μg/ml bovine serum albumin), supplemented with DNA polymerase 1 (5 U), 1 mM ATP, and T4 DNA ligase (10 U). For control experiments, the repair enzyme cocktail was not added to the reaction buffer. The enzymes were purchased from New England Biolabs (USA). The reaction was terminated with 2.5 μl of 0.25 M EDTA. The samples were stored at 4 °C prior to analysis.

### Silanization of coverslips

The functionalization of glass coverslips was adapted from Wei et al. [46]. Briefly, standard 22 × 22 mm, No. 1 coverslips were submerged in a mixture of 1% (3-aminopropyl) triethoxysilane (APTES, Sigma), 1% allyltrimethoxysilane (ATMS, Sigma), in acetone, and coated for 1 h. After the completion of silanization, the coated coverslips were rinsed with three cycles each of acetone and milli-Q water and then dried by air purging. The air-dried coverslips were stored at room temperature in a parafilm tight petridish and used within 3 days.

### DNA staining and imaging

2 μl of the fluorescently labeled DNA was stained with 320 nM YOYO-1 (Invitrogen) and 2 μl of β-mercaptoethanol (BME, Sigma-Aldrich) in a total volume of 100 μl of 0.5 × TBE. Then, 3.8 μl of each sample was extended on a 22 × 22 mm coverslip by placing the solution at the interface of an activated coverslip and a clean microscopy slide (VWR Frosted). The extended DNA molecules were imaged with a fluorescence microscope (Zeiss Observer.Z1) using an Andor iXON Ultra EMCCD camera. The images were captured using 475/40 and 640/30 band-pass excitation filters and 530/50 and 690/50 bandpass emission filters, for YOYO-1 and Aminoallyl-dUTP-ATTO-647 N, respectively. EM gain setting of 100 and exposure times of 10 ms and 500 ms were used for YOYO-1 and Aminoallyl-dUTP-ATTO-647 N, respectively.

### Data analysis

The microscopy images were analyzed using a custom-made software [[47], [48], [49]]. The total DNA length of each molecule was estimated and the number of colocalized Aminoallyl-dUTP-ATTO-647 N spots on each molecule was counted. The total number of ATTO-647N spots in an image set was divided by the total DNA length in pixels to get the ratio of damage sites/length and finally converted into sites/M base pair (MBp) (1 pixel = 0.129 μm). The value for converting sites/length to sites/MBp of DNA was calculated by analyzing lambda DNA (48,502 base pairs) in similar buffer conditions (1 μm = ~3000 bp). In total, 16–18 MBp of DNA was used for analysis for each sample.

The formula for calculating residual DNA damage:

$$\text{Residual}\;\mathrm{DNA}\;\text{damage}\%=\left(\text{Sites}/\mathrm{MBp}\;\left(90\;\min\;\text{post}-\text{irradiation}\right)-\text{Sites}/\mathrm{MBp}\;\left(\text{Untreated}\right)\right)/\left(\text{Sites}/\mathrm{MBp}\;\left(0\;\min\;\text{post}-\text{irradiation}\right)-\text{Sites}/\mathrm{MBp}\;\left(\text{Untreated}\right)\right)\times 100$$

### Statistics

The data was collected from three healthy volunteers on three different days and the corresponding standard deviation is reported. Two tailed- *t*-tests were performed using excel, *p*-values were determined and p < 0.05 was considered to be statistically significant. ** represents p < 0.01 and * represents p < 0.05 as measured by two tailed t-tests assuming equal variance.

## Results

**Fig. 1:**
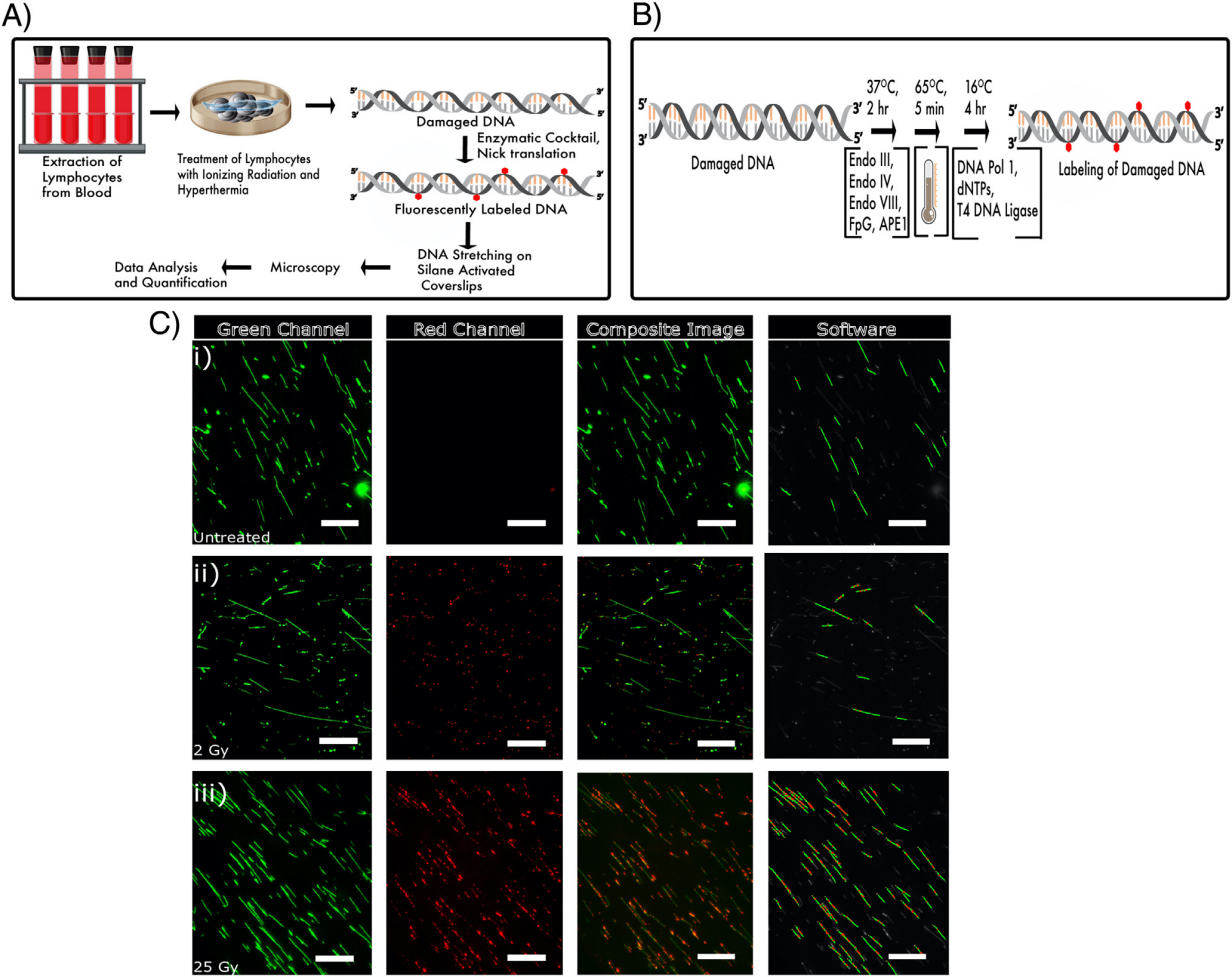
(A) Schematic outline of sample preparation and data collection for detection of DNA damage in PBMCs from healthy donors. (B) Schematic outline of DNA damage labeling in presence of an enzyme cocktail. (C) (i) Untreated (ii) 2 Gy treated and (iii) 25 Gy treated PBMCs, which have been labeled with fluorescent nucleotides after prior treatment with the enzyme cocktail. The DNA backbone was stained with YOYO-1 (green channel) and damage sites were labeled with Aminoallyl-dUTP-ATTO-647 N (red channel). Scale bar = 25 μm. (For interpretation of the references to colour in this figure legend, the reader is referred to the web version of this article.)

The assay for detecting single-strand lesions formed by IR and hyperthermia is schematically outlined in Fig. 1A. PBMCs were prepared and treated with IR and/or hyperthermia. The repair of the extracted, damaged DNA was initiated with a cocktail of repair enzymes, leading to fluorescently labeled DNA where each damage site is labeled with a fluorescent spot. A schematic of the steps involved in the labelling is shown in Fig. 1B. To visualize the spots, the DNA was stretched on functionalized glass slides and the DNA backbone was stained with the fluorescent dye YOYO-1. Representative images acquired from the microscope as well as data quantification is depicted in Fig. 1C. The spots are counted and reported as the number of damage sites/MBp of stretched DNA.

### Effect of APE1 on detection of IR induced DNA damage

**Fig. 2:**
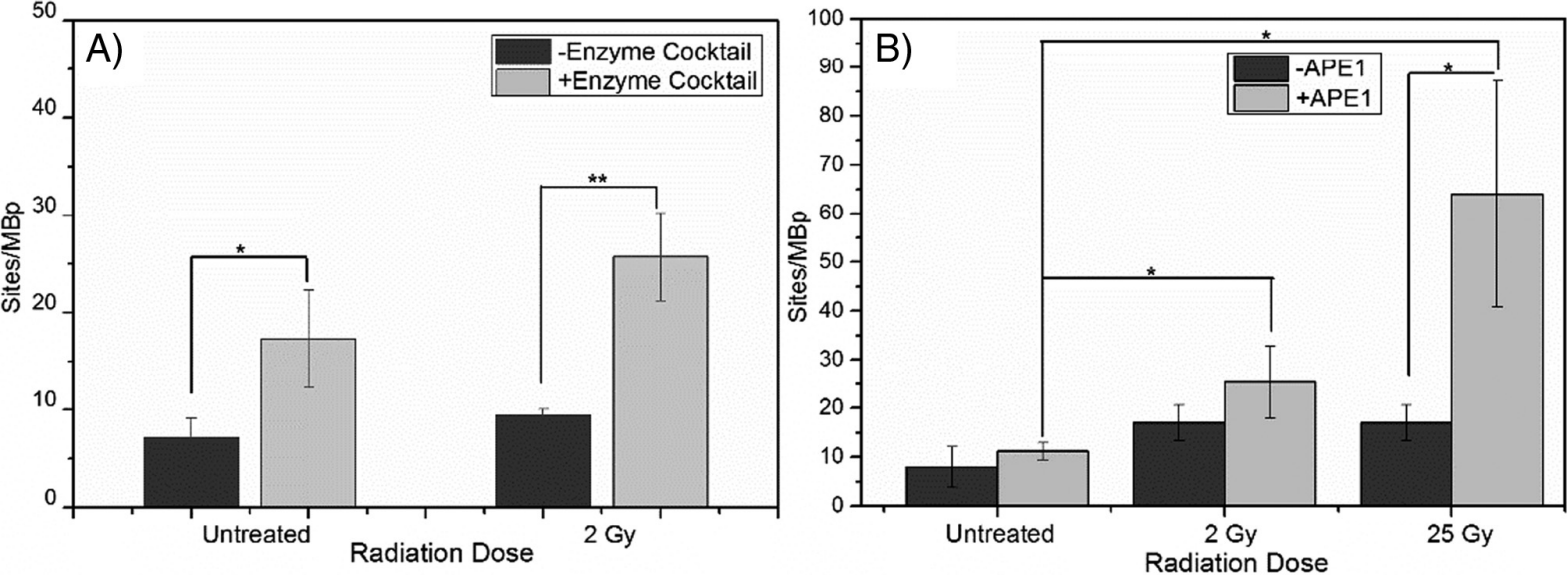
A) DNA damage detected in PBMCs with (gray) and without (black) an enzyme cocktail consisting of APE1, FpG, Endo III, Endo IV, Endo VIII. DNA polymerase 1 and T4 DNA ligase are used for all samples. Each value represents mean ± SD. ** represents p < 0.01 and *p < 0.05 as measured by two tailed *t*-tests with equal variance from three separate experiments. B) DNA damage detected in PBMCs irradiated with IR doses of 0 Gy, 2 Gy and 25 Gy, without (dark gray) and with (light gray) the APE1 enzyme in the enzyme cocktail. Each value represents mean ± SD. *p < 0.05 as measured by two tailed t-tests with equal variance from three separate experiments.

Using our single-molecule DNA-damage assay we first quantified the damage for PBMCs treated with 2 Gy of IR using only DNA polymerase 1 and T4 DNA ligase and a ~32% increase in DNA damage was detected compared to control samples (Fig. 2A). This indicates that only a limited fraction of the damage sites are simple DNA nicks, which can be repaired by DNA polymerase 1 alone. We next used a cocktail of DNA repair enzymes (see methods), with or without APE1. Using both cocktails, we detected ~17–64 sites/MBp in the IR treated samples in comparison to the untreated sample where ~8–11 sites/MBp were detected (Fig. 2B), indicating the presence of a significant fraction of DNA lesions that cannot be nick-translated with only DNA polymerase 1 but can be repaired by the enzymatic cocktails. The inclusion of APE1 increased the number of detected single-strand lesions slightly at 2 Gy and significantly at 25 Gy (~3.8 times higher than without APE1). Thus the APE1 enzyme significantly improves the detection of IR-induced DNA damage, in particular at higher doses. Fig. S1 shows a closer analysis of lower doses of radiation that are clinically relevant and we see a steady increase with increasing IR dose.

### Repair kinetics of IR-induced damage

**Fig. 3:**
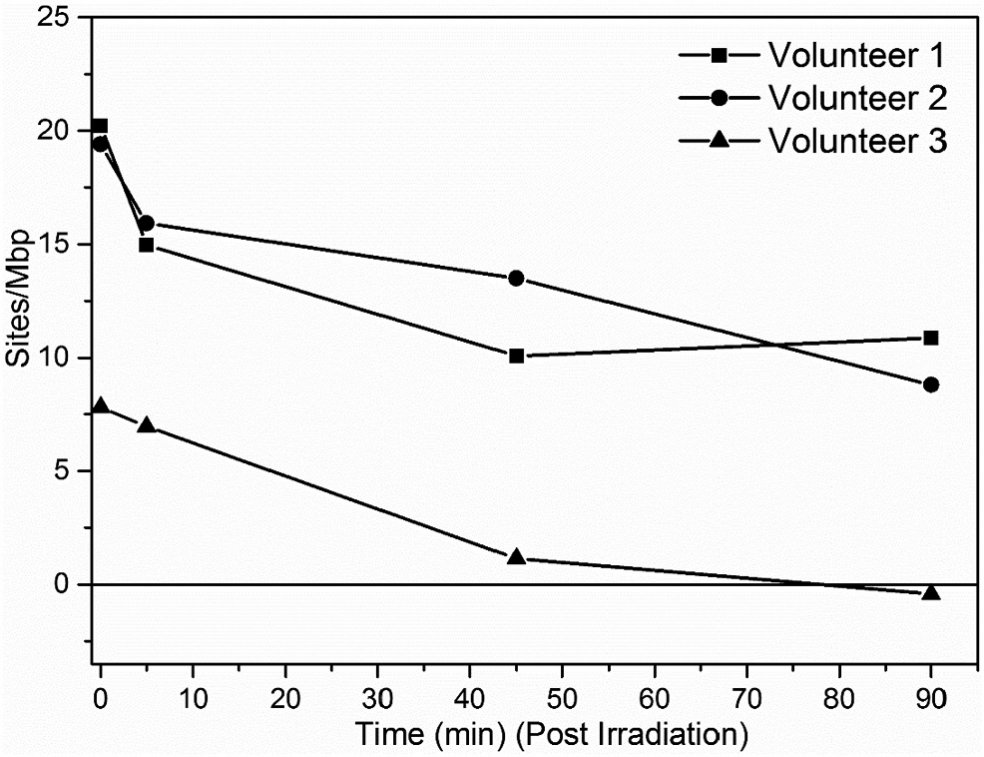
DNA damage repair in PBMCs irradiated with 2 Gy of IR and incubated at 37 °C in RPMI medium for 0 min to 90 min in three healthy volunteers. The damage level before treatment has been subtracted from all data points.

The assay has previously been used to measure DNA repair kinetics for UV and H_2_O_2_ induced damage [36]. To measure repair kinetics of IR induced DNA-damage we incubated PBMCs from healthy donors (n = 3) for different times at 37 °C after a 2 Gy IR dose (Fig. 3). The repair is similar for all three samples, but the remaining amount of damage after 90 min differs significantly. Both these characteristics can be retrieved independently using this assay.

### Effect of hyperthermia on the formation of single-strand damage caused by IR

**Fig. 4:**
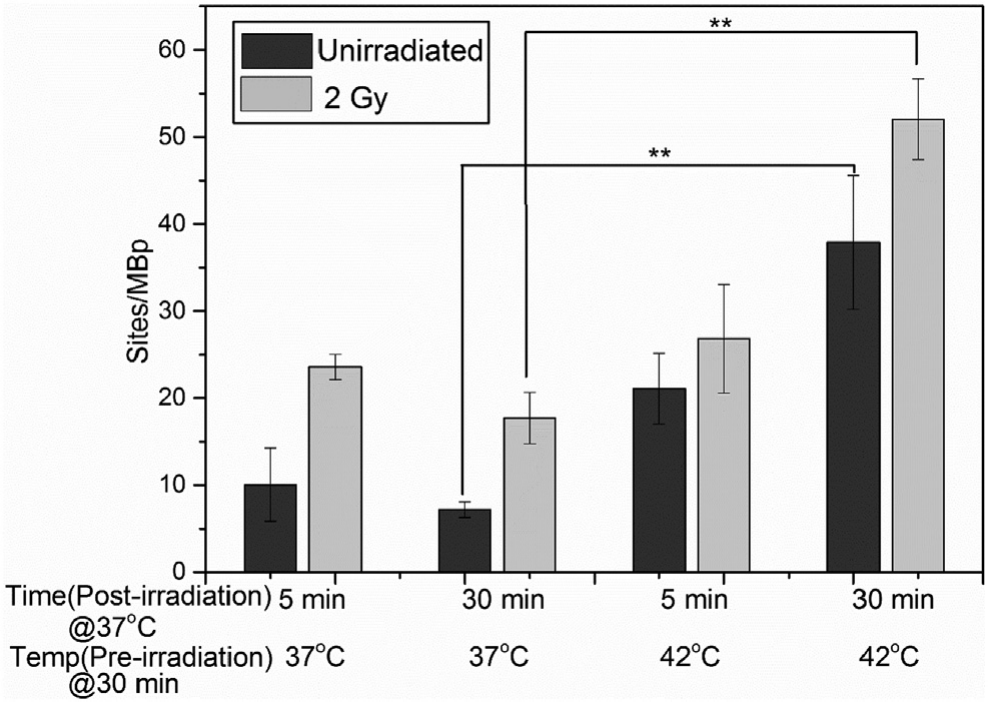
DNA damage in PBMCs incubated at 37 °C or 42 °C before IR treatment at 2 Gy (gray) followed by post-incubation at 37 °C, controls in black. The unirradiated samples were maintained at 37 °C. Each value represents mean ± SD. ** represents p < 0.01 as measured by two tailed t-tests with equal variance from three separate experiments.

Hyperthermia is an emerging treatment used in combination with IR and/or chemotherapy to sensitize cells to the treatment [13,50]. We first established the amount of damage for PBMCs only exposed to hyperthermia (incubation at 42 °C for 30 min). Immediately after hyperthermia treatment, a 29% increase in the amount of damage was detected compared to control samples kept at 37 °C (Fig. S2, Supporting Information). However, if the cells were further incubated at 37 °C after the hyperthermia treatment, the amount of damage increased ~2 fold after 5 min and ~4 fold after 30 min, respectively, compared to corresponding untreated samples (preincubation temperature 37 °C) (Fig. 4). In PBMCs treated with hyperthermia followed by IR, an additive increase in the level of DNA damage was detected. Interestingly, hyperthermia treatment prior to IR treatment changes the repair trajectory of IR induced DNA damage. In case of only IR treatment, the repair of single-strand lesions was initiated as is evident from the ~1.3 fold decrease in lesions detected after 30 min of incubation in RPMI medium. In contrast, hyperthermia treatment before administration of IR the amount of single-stranded DNA damage increased ~2 fold after 30 min.

## Discussion

In this study we report the adaptation of a previously reported assay [36] for directly quantifying IR-induced DNA damage in PBMCs isolated from patient blood samples. We show that inclusion of APE1 in the enzyme cocktail used significantly increased the amount of DNA lesions detected after IR treatment. The use of the APE1 enzyme was particularly useful in the detection of DNA damage at higher doses of IR. APE1 is the major end processing enzyme involved in BER, having abasic site-specific 5′endonucleolytic activity, 3′phosphodiesterase, 3′phosphoglycolate, 3′phosphatase and 3′-5′exonuclease capabilities [51,52]. Although, through our assay we demonstrated the importance of APE1 in lesion detection, our data also demonstrate that other endonucleases and glycosylases used in the assay are important constituents of the enzyme cocktail. Future studies with single enzymes and different enzyme combinations are required to add additional information on the role of each enzyme in the repair of IR and hyperthermia induced damage. In the enzymatic cocktail we used both Endo IV and APE1 which act on similar types of DNA lesions but under our experimental conditions, APE1 provided additional processing of the IR damaged samples when used with other enzyme cocktail members at higher IR dose.

Patients with deficiencies in DNA repair genes are known to be sensitive to DNA damaging agents, including IR [53]. Furthermore, XRCC1, XRCC3, and ATM gene-variants have been correlated with hypersensitivity towards radiotherapy [[54], [55], [56]]. Using this modified assay, it was possible to quantify IR induced damage in different individuals and establish the repair kinetics, using measurements of the change in the amount of damage over time. The repair profile of the IR-treated DNA in this study is similar to that previously reported using comet assay [57], confirming that the modified assay is a possible alternative to the comet assay for the measurement of IR induced damage and repair in patients. In the above mentioned study, in case of IR treated healthy volunteers, residual DNA damages was varying in different individual from none to 30% [57], which is in agreement with the variation in residual DNA damage observed in our study.

We also combined IR and hyperthermia, a joint therapy that is making its way into the clinical setting [58]. Several different mechanisms have been suggested for radiosensitization of tumors by hyperthermia. Physiologically, pre-heating the tumour, prior to radiotherapy effects the blood flow and the microenvironment of the tumour [59]. On a molecular level, hyperthermia has been shown to inhibit DNA repair *via* both homologous recombination [60] and non-homologous end joining [61]. Additionally, some studies suggest that hyperthermia can induce DNA damage on its own [20,62]. Most studies of the molecular mechanism of radiosensitization by hyperthermia have used tumour cell lines but PBMCs more closely model normal tissue damage induced by hyperthermia. PBMCs are non-dividing cells which may respond differently to DNA damage relative to the rapidly dividing tumour cells. Interestingly, we observed an increase in DNA damage with hyperthermia alone, which continued to rise after the sample was placed at 37 °C for up to 30 min. This finding is in agreement with a previous report on white blood cells showing that hyperthermia led to a rapid increase in the number of strand breaks during subsequent incubation of 37 °C [19]. When combining IR and hyperthermia, the DNA damage was additive, and no repair was detected 30 min post-irradiation which may be due to hyperthermia-dependent inhibition of DNA repair post-IR as reported by others [12].

Using this assay to measure DNA damage has several advantages relative to previously reported methods. Firstly, the assay can be modified to measure different types of DNA damage and the enzymes can be chosen so that specific types of damage can be quantified, as has been shown in a recent paper by Torchinsky et al. [48]. The comet assay can also, to some extent, be modified to label specific DNA damage types [63]. Furthermore, the assay allows direct investigation of heterogeneities in the amount of DNA damage along the genome. By combining the damage assay with optical DNA mapping it is possible to identify where along the genome each damage site is located [64]. This could be combined with commercial systems for optical DNA mapping to investigate genome-wide levels of DNA damage, similar to what was recently demonstrated for the epigenetic markers 5-methylcytosine [65] and 5-hydroxymethylcytosine [66].

If the assay is shown to also be able to detect defective DNA repair in patients, it has the potential to be used clinically for personalized radiation- and chemotherapy. To reach this goal, the assay needs to be validated using individuals with known DNA damage sensitivities, as well as resistant and sensitive cell lines. Further, the repair profiles of cancer patients' PBMCs using the assay needs to be correlated to therapy response. In this study, blood is used as a source of lymphocytes, which is relatively easily obtained and allows investigation of differences in the normal tissue response among individuals. This also enables studying patients undergoing chemotherapy to evaluate whether their cell response correlates with the effectiveness of the therapy. However, it should be taken into consideration that the DNA damage response in normal cells does not reflect that of tumour tissue, or other tissues from the individual.

To conclude, the present work demonstrates the development of an enzymatic assay that determines the total number of single-strand lesions generated on DNA, tailored to sense IR and hyperthermia induced lesions. The enzyme APE1 was included in the enzyme cocktail to increase the number of detected lesions, in particular at high IR doses, where clustered DNA damage is common. The marked increase seen in the number of single-strand lesions due to hyperthermia and IR can be explained by both inhibition of DNA repair and formation of more single-strand lesions. The results from this study correlate with prior studies in the field of hyperthermia and IR-based therapy, indicating that the assay is robust. We propose that the assay could be used to identify patients with deficiencies in repair of IR-induced damage, which would be a way of personalizing IR treatment for patients, that in turn would minimize side effects for sensitive patients.

## Author contribution

Vandana Singh: Conceptualization, Methodology, Validation, Formal Analysis, Investigation, Writing - Original draft preparation. Funding Acquisition. Pegah Johansson: Conceptualization, Resources, Writing - Reviewing and Editing, Supervision. Dmitry Torchinsky: Software, Formal Analysis. Yii-Lih Lin: Software. Robin Öz: Methodology. Yuval Ebenstein: Conceptualization, Methodology, Software, Formal Analysis. Ola Hammarsten: Conceptualization, Resources, Writing - Reviewing and Editing, Supervision, Funding Acquisition. Fredrik Westerlund: Conceptualization, Methodology, Writing - Reviewing and Editing, Supervision, Project Administration, Funding Acquisition.

## Appendix A Supplementary data

Supplementary figures Image 1
