# Supplementary material for: Quantifying DNA damage induced by ionizing radiation and hyperthermia using single DNA molecule imaging
Source: Transl Oncol. 2020 Jul 8;13(10):100822. doi: 10.1016/j.tranon.2020.100822 (PMC7350159; doi:10.1016/j.tranon.2020.100822)
Supplement: Supplementary file 1 — Supplementary figures [file mmc1.docx]

Appendix A. Supplementary data

*Fig S1. DNA damage detected in PBMCs irradiated with IR ranging from 0 Gy - 2.5 Gy and treated with an enzyme cocktail consisting of APE1, FpG, Endo III, Endo IV, Endo VIII, DNA polymerase 1, T4 DNA ligase. Each value represents mean±SD. ** represents p<0.01, * p<0.05 measured by two tailed t-tests with equal variance from three separate experiments.*

*

*

*Fig S2. DNA damage detected in PBMCs kept at temperatures ranging from 4°C to 42°C for 30 min. Each value represents mean ±SD. *p<0.05 by two tailed t-tests with equal variance from three separate experiments.*
